# CaMKII regulates the strength of the epithelial barrier

**DOI:** 10.1038/srep13262

**Published:** 2015-08-18

**Authors:** Ryo Shiomi, Kenta Shigetomi, Tetsuichiro Inai, Masami Sakai, Junichi Ikenouchi

**Affiliations:** 1 Department of Biology, Faculty of Sciences, Kyushu University, Fukuoka 812-8581, Japan; 2 Department of Morphological Biology, Fukuoka Dental College, 2-15-1 Tamura, Sawara-ku, Fukuoka 814-0193, Japan; 3 PRESTO, Japan Science and Technology Agency, Saitama 332-0012, Japan

## Abstract

Epithelial cells define the boundary between the outside and the inside of our body by constructing the diffusion barrier. Tight junctions (TJs) of epithelial cells function as barriers against invasion of harmful microorganisms into the human body and free diffusion of water or ions from the body. Therefore, formation of TJs has to be strictly controlled in epithelial cells. However, the molecular mechanisms governing this regulation are largely unknown. In this study, we identified Ca^2+^/calmodulin-dependent protein kinase II (CaMKII) as a regulator of the barrier function of TJs. CaMKII inhibition led to enlargement of TJ-areas and up-regulation of the barrier function. CaMKII inhibition induced excess TJ formation in part by the activation of AMP-activated protein kinase (AMPK) and subsequent phosphorylation of claudin-1. As up-regulation of epithelial barriers is essential for the prevention of chronic inflammatory diseases, the identification of CaMKII as a modulator of TJ function paves the way for the development of new drugs to treat these diseases.

Although the molecular components of tight junctions (TJs) were discovered^1^,^2^,^3^, it remains largely unclear how the formation of TJs is regulated. In most epithelial cells, TJs are formed only at the most apical part of lateral membranes. However, it is reported that TJs are newly formed in the basolateral region of Sertoli cells during spermatocyte translocation across Sertoli cells, when spermatocytes become enclosed within a network of transient compartments bounded by apical and basal TJs^4^. TJs become enlarged under hyperosmotic stress^5^; therefore, epithelial cells likely have mechanisms regulating the amount of TJs. In pathological conditions such as inflammatory bowel diseases, asthma or atopic dermatitis, two consequences result from the impairment of the epithelial barrier: leakage and flux of ions or water from internal compartments of the body and a facilitated uptake of noxious antigens from outside the body. Thus, it is clinically important to understand the molecular mechanisms involved in TJ formation^6^,^7^,^8^,^9^. If the molecular mechanisms of TJ regulation are revealed, enhancing the epithelial barrier will become a possible therapeutic approach to treat inflammatory bowel diseases, asthma and atopic dermatitis.

It is now well-established that claudins, proteins with four transmembrane domains, are essential for TJ formation. A previous study revealed that over-expression of claudin is enough to reconstitute ectopic TJ formation in L fibroblasts^10^. However, in epithelial cells, forced expression of claudins is not sufficient for the up-regulation of the barrier function, suggesting the existence of molecular mechanisms that prevents surplus TJ formation in these cells.

Polymerization of claudins into TJ strands is likely strictly regulated. One important regulator of TJ formation is the ZO family of proteins. We and others have revealed that the ZO family of proteins is essential for TJ formation^11^,^12^,^13^,^14^. We previously demonstrated that forced dimerization of ZO-1 induces polymerization of claudins and aberrant TJ strands, suggesting that ZO-1 is a nucleator of claudins^13^,^14^. Therefore, ZO-1 determines where claudins should polymerize into TJs along the apico-basal axis of epithelia.

In addition to being regulated by the ZO family of proteins, excess claudin-1 should be removed from the plasma membrane to prevent spontaneous polymerization of claudins into extra TJs in epithelial cells. Although unpolymerized claudins are retained at the basolateral membrane in cultured epithelial cells and in tissues, extra TJs are not formed at the basolateral membrane domains. To prevent spontaneous polymerization of claudins and unnecessary TJ formation, the concentration of unpolymerized claudins should be kept under the threshold of spontaneous polymerization. Thus, molecular mechanisms to control the concentration of unpolymerized claudins at the plasma membrane exist, but have yet to be clarified.

In the present study, we revealed that CaMKII inhibition led to enlargement of TJ areas and up-regulation of epithelial barrier function. These effects were owed to increased junctional contractility and decreased mono-ubiquitination of claudin-1.

## Results

### Identification of KN-93 as a TJ-formation promoting agent

**Figure 1:**
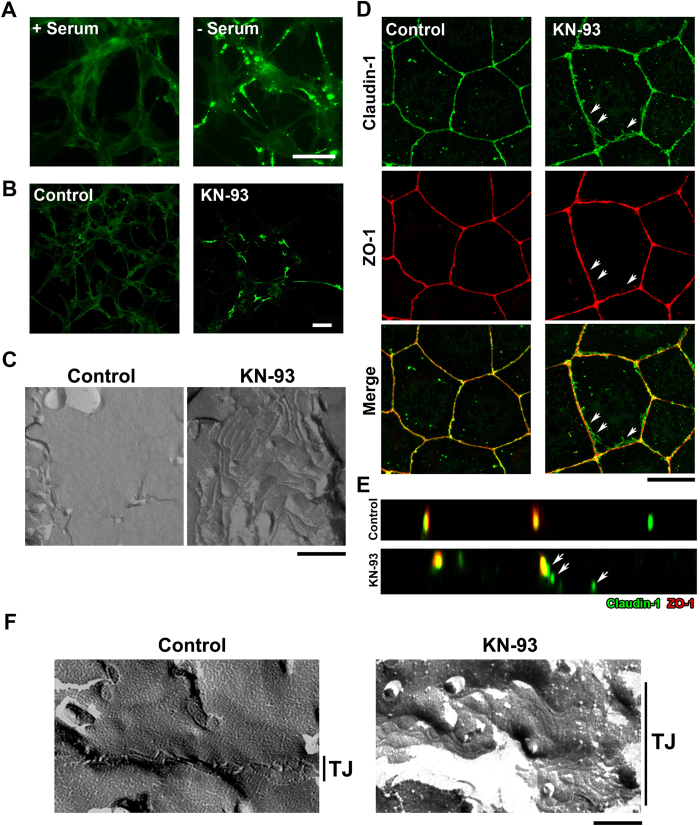
KN-93, a CaMKII inhibitor, induces enlargement of TJs (**A**) GFP-claudin-1 accumulated at cell-cell contacts only in the serum starved condition for 24 hours in GFPmCL1L cells. Scale bar, 5 μm. (**B**) Treatment with 10 μM KN-93 for 5 hours induced TJ formation in GFPmCL1Lcells. Scale bar, 10 μm. (**C**) Freeze-fracture images of cell contact planes of GFPmCL1L cells treated with DMSO (control) (left) and GFPmCL1L cells treated with KN-93 for 5 hours (right). Scale bar, 500 nm. (**D**,**E**) Treatment with 10 μM KN-93 for 5 hours induced excess TJ formation at basolateral membrane regions in CSG1 cultured epithelial cells. Cells treated with DMSO (control) or 10 μM KN-93 were doubly stained with anti-claudin-1 pAb (green) and anti-ZO-1 mAb (red). Note that these extra TJ networks did not contain ZO-1 (arrows). Scale bar, 10 μm. (**F**) Freeze-fracture images of TJ strands of EpH4 cells treated with DMSO (control) (left) and EpH4 cells treated with KN-93 for 6 hours (right). Scale bar, 200 nm.

We first established several clones of L fibroblasts expressing GFP-tagged mouse claudin-1 (GFPmCL1L) at various levels. We isolated the GFPmCL1L cell clone, which had a low expression level at which GFP-claudin-1 was unable to form TJs, but localized entirely to the plasma membrane (Fig. 1A). Unexpectedly, we found that TJ formation occurred in GFPmCL1L cells under serum starved conditions, as determined by the enrichment of GFP signal at the cell-cell contacts (Fig. 1A). As this TJ formation under serum starved conditions might be promoted by a cell signaling pathway, we performed drug screening to identify these signaling pathways.

We examined the effects of approximately 400 bioactive chemical regents on the polymerization of GFP-claudin-1 in GFPmCL1L cells. We identified KN-93, a specific CaMKII inhibitor^15^, as a potent inducer of spontaneous polymerization of GFP-claudin-1 at cell-cell contacts in GFPmCL1L cells (Fig. 1B). We confirmed formation of TJ strands of polymerized GFP-claudin-1 at the plasma membrane using freeze-fracture electron microscopy (Fig. 1C).

**Figure 2:**
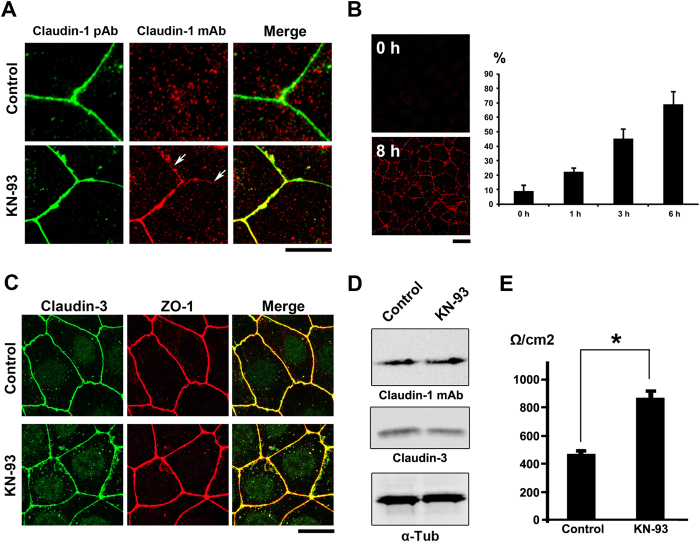
KN-93 induces up-regulation of barrier function (**A**) Claudin-1 mAb (Clone 2H10D10) recognized claudin-1, which contributed to the formation of ectopic TJs induced by the treatment with KN-93 (arrows). Scale bar, 5 μm. (**B**) Quantification of the time-dependent effect of KN-93 on the enlargement of TJs. EpH4 cells treated with KN-93 for the indicated times were stained with DAPI and the anti-claudin-1 mAb (2H10D10). The number of cells with extended TJs was determined as a percentage of the total number of cells (based on DAPI staining) in the same field. Each experiment (n = 3) included 100 cells. Data are represented as the mean ± s.d. (**C**) EpH4 cells were treated with DMSO (control) or 10 μM KN-93 for 5 hours and stained with anti-claudin-3 pAb (green) and anti-ZO-1 mAb (red). Scale bar, 20 μm. (**D**) Immunoblotting of whole-cell lysates from EpH4 cells treated with DMSO (control) or 10 μM KN-93 with the indicated antibodies. (**E**) CSG1 cells were cultured in Transwell chambers and treated with DMSO (control) or 10 μM KN-93 for 5 hours and then analyzed for their TER. Analysis of TER showed about a 1.8-fold increase after the treatment with KN-93. Data are mean ± s.d.; *P < 0.05 (Student’s t-test).

We next examined whether KN-93 induces enlargement of TJs in epithelial cells. When compared with the staining pattern of endogenous claudin-1, treatment with KN-93 induced the formation of laterally enlarged TJ networks in cultured epithelial cell lines, such as CSG1 cells (Fig. 1D,E and S1) or EpH4 cells (Fig. 2C). Surplus TJs induced by KN-93 did not contain undercoat proteins of TJs such as ZO-1 (Fig. 1D,E, arrowheads). This suggests that treatment with KN-93 enlarge TJs without affecting normal polarized distribution of ZO-1. We confirmed KN-93 treatment increased the number of TJ strands (Fig. 1F). Enlargement of TJs was also induced by treatment of GFPmCL1L or CSG1 cells with another CaMKII inhibitor, namely Autocamide-2-related Inhibitory Peptide (Fig. S2A).

To distinguish laterally enlarged TJ areas from the normal TJ strands, we used anti-claudin-1 mAb (clone 2H10D10). The 2H10D10 antibody was able to detect excess TJs formed by treatment of CSG1 cells with KN-93 (Fig. 2A). The epitope of this antibody was masked by binding of ZO-1 (K.S. and J.I., unpublished observation), indicating that it preferentially recognized claudin-1 that was not bound to ZO-1.

Taking advantage of this unique character of the mAb, we quantified the effect of KN-93 treatment on the formation of enlarged TJs (Fig. 2B). Immunostaining with the anti-claudin1 mAb (2H10D10) revealed a time-dependent effect of KN-93 on the number of cells with enlarged TJs (Fig. 2B).

These enlarged TJ networks contained other TJ membrane proteins such as claudin-3 (Fig. 2C). KN-93 treatment did not change the protein expression levels of claudin-1 or claudin-3 (Fig. 2D).

We next investigated the effects of KN-93 on the epithelial barrier function by measuring trans-epithelial resistance (TER) (Fig. 2E). After treatment with KN-93 for 3 hours, the TER value significantly increased in CSG cells (871.3 ± 46.2 **Ω**/cm2) compared with that of control cells (469.6 ± 23.5 **Ω**/cm2). We therefore concluded that KN-93 is a potent TJ-formation promoting agent. In the following experiments, we further investigated how treatment with KN-93 up-regulated the epithelial barrier.

### CaMKII inhibition enhances epithelial barrier function through up-regulation of junctional contractility

**Figure 3:**
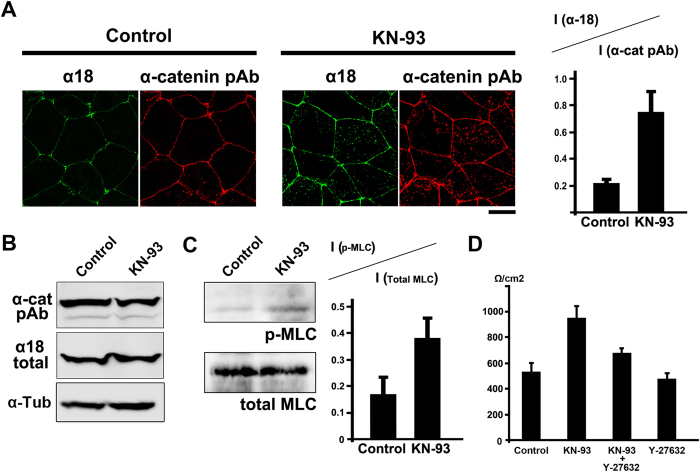
KN-93 treatment enhances epithelial barrier function by increasing junctional contractility (**A**) EpH4 cells were treated with DMSO (control) or 10 μM KN-93 for 5 hours and stained with α18 antibody, which recognizes a tension-dependent conformation of α-catenin, and anti-α-catenin pAb. In control cells, α18 antibody stained preferentially tricellular corners of epithelial cells (arrowheads). In KN-93 treated cells, α18 antibody strongly stained bicellular cell-cell junctions (arrows). Scale bar, 20 μm. The ratio of the fluorescence value of the staining of the α18 antibody to the fluorescence value of the staining of the α-catenin polyclonal antibody at the bicellular junctions was calculated. Data are mean ± s.d.; *P < 0.05 (Student’s t-test). (**B**) Immunoblotting of whole-cell lysates of EpH4 cells treated with DMSO (control) or 10 μM KN-93 with the indicated antibodies. (**C**) Immunoblotting of whole-cell lysates of EpH4 cells treated with DMSO (control) or 10 μM KN-93 with the indicated antibodies. The ratio of phosphorylated MLC to total MLC was quantified in EpH4 cells treated with DMSO (control) or 10 μM KN-93. Data are mean ± s.d.; *P < 0.05 (Student’s t-test). (**D**) EpH4 cells were cultured in Transwell chambers and treated with DMSO (control), 10 μM KN-93, 10 μM KN-93 and 10 μM Y-27632 or 10 μM Y-27632 for 5 hours and then analyzed for their TER. The TER values in this experiment were as follows: control 533.6 ± 67.2 **Ω**/cm^2^, KN-93 953.0 ± 93.0 **Ω**/cm^2^, KN93 + Y27632 679.3 ± 35.4 **Ω**/cm^2^, Y-27632 424.3 ± 66.4 **Ω**/cm^2^. Data are mean ± s.d.; *P < 0.05 (Student’s t-test).

We looked at changes in the distribution of cell adhesion molecules other than claudin-1 after treatment with KN-93. Among the cell adhesion molecules tested, the intensity of staining with the α18 antibody, which recognizes a tension-dependent conformation of α-catenin^16^, differed between KN-93- and DMSO-treated cells (Fig. 3A). In KN-93 treated cells, the α18 antibody stained bicellular adherens junctions strongly, suggesting that junctional contractility was up-regulated along the entire circumferential ring in these cells (Fig. 3A). We quantified the ratio of the fluorescence value of the α18 antibody staining to the fluorescence value of the α-catenin polyclonal antibody staining at the bicellular junctions. The protein expression level of α-catenin was unchanged by the KN-93 treatment (Fig. 3B). These results indicate treatment with KN-93 significantly enhanced the junctional contractility. In agreement with these observations, we used western blotting to show phosphorylation of myosin light chain 2 (MLC-2) was significantly increased after the treatment with KN-93 (Fig. 3C).

We next reasoned that up-regulation of junctional contractility may be involved in the increase of barrier function induced by treatment with KN-93. Inhibition of rho-kinase by treatment with Y-27632 abrogated the increase of TER by KN-93-induced CaMKII inhibition, indicating that up-regulation of junctional contractility is necessary for the enhanced epithelial barrier function we saw with KN-93 treatment alone (Fig. 3D).

Taken together, these results demonstrated that CaMKII inhibition leads to up-regulation of junctional contractility, which in turn contributes to the strengthening of epithelial barrier function.

### CaMKII inhibition leads to AMPK activation

In addition to the increased junctional contractility, CaMKII inhibition by KN-93 caused enlargement of the TJ-containing areas. We previously demonstrated that ZO family proteins determined where claudin-1 should be polymerized as forced dimerization of ZO-1 was sufficient to induce ectopic TJ formation at the lateral membrane^14^. The laterally enlarged TJs induced by treatment with KN-93 did not contain any ZO family undercoat proteins, indicating another molecular mechanism likely regulates the assembly of claudin-1 at the plasma membrane (Fig. 1D). As mentioned previously, the enlargement of TJ networks decreases transportation of various materials across the epithelial sheets through the paracellular pathway^17^,^18^,^19^. Therefore, we next investigated the molecular mechanisms by which TJ areas were enlarged when cells were treated with KN-93.

**Figure 4:**
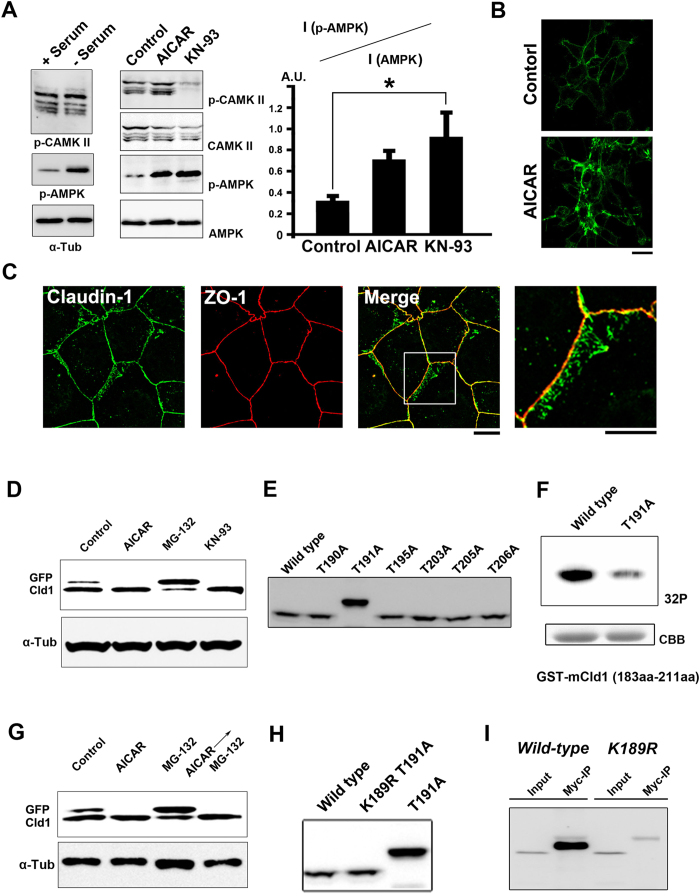
Inhibition of CaMKII leads to AMPK activation and phosphorylation of claudin-1 (**A**) Immunoblotting of whole-cell lysates from GFPmCL1L cells treated with normal medium, serum-depleted medium, DMSO (control), 1 mM AICAR or 10 μM KN-93 with the indicated antibodies. Note that treatment with KN-93 dramatically increased the active form of AMPK. The ratio of the active form to total AMPK was quantified. Data are mean ± s.d.; *P < 0.05 (Student’s t-test). (**B**) Activation of AMPK by the treatment with 1 mM AICAR for 24 hours induced accumulation of GFP-claudin1 at cell-cell contacts in GFPmCL1L cells. Scale bars, 10 μm. (**C**) Activation of AMPK by the treatment with 1 mM AICAR for 24 hours induced ectopic TJs in EpH4 cells. Scale bars, 10 μm. (**D**) GFP-claudin-1 migrated as a doublet, and the slower migrating band was reduced by the treatment with 10 μM KN-93. (**E**) HEK293 cells were transfected with N-terminally GFP-tagged wild-type claudin-1, or the T190A, T191A, T195A, T203A, T205A, or T206A mutants. The cell lysates were immuno-blotted with an anti-GFP antibody. (**F**) AMPK phosphorylates the cytoplasmic tail of claudin-1 as determined by an *in vitro* kinase assay. GST-claudin-1 COOH-terminal tail was incubated with recombinant AMPK in the presence of [γ-^32^P] ATP. The phosphorylated proteins were resolved by SDS/PAGE and detected by autoradiography. (**G**) The upper band of GFP-claudin-1 was decreased by the treatment with 1 mM AICAR, whereas the upper band was increased by the treatment with 10 μM MG-132. AMPK activation prior to proteasome inhibition reduced the upper band. (**H**) HEK293 cells were transfected with N-terminally GFP-tagged wild-type claudin-1, the K189R/T191A mutant and the T191A mutant. The cell lysates were immuno-blotted with anti-GFP antibody. (**I**) Claudin-1 was mono-ubiquitinated at Lys-189. Cells were transfected with GFP-claudin-1 or GFP-claudin-1 K189A mutant and myc-ubiquitin. The cell lysates were subjected to immunoprecipitation with anti-myc antibody, followed by immunoblotting with anti-GFP antibody.

Owing to our observation that TJs were formed in serum starved GFPmCL1L cells at the initial stage of this study, we wondered if CaMKII activity is down-regulated by serum starvation. As the activated CaMKII further autophosphorylates at Thr-286 to be active^20^, we examined the changes in the phosphorylation level of CaMKII using phospo-CaMKII (Thr286) antibody by serum starvation. We found that phosphorylation of CaMKII was not changed by serum starvation (Fig. 4A). As AMPK, an energy-sensing kinase was reported to be activated under serum starvation^21^, we wondered if the activation of AMPK caused by serum starvation is involved in the TJ formation in GFPmCL1L cells.

A relationship between the inhibition of CaMKII and the activation of AMPK had not been shown previously, so we examined whether crosstalk existed between these two kinases in an attempt to narrow down the signaling pathway(s) that control TJ formation. Although the molecular mechanisms are not clear at present, we found that CaMKII inhibition strongly activated AMPK, but AMPK activation by AICAR treatment did not induce CaMKII inhibition (Fig. 4A). When GFPmCL1cells or EpH4 cells were treated with AICAR, extra TJ formation was observed in both cell lines (Fig. 4B,C). Contrary to the treatment with KN-93, AMPK activation only was not sufficient to enhance epithelial barrier function, as we found the TER value was not significantly changed after treatment with AICAR (Fig. S2B). The staining pattern of α18 antibody did not change after treatment with AICAR (Fig. S2C), suggesting that junctional contractility was unaffected by treatment with AICAR. Therefore, we reasoned that activation of AMPK caused by the treatment with AICAR or under serum starvation promoted the surplus formation of TJs without affecting junctional contractility.

In previous studies, two groups reported that AMPK is activated during Ca^2+^ -induced TJ assembly^22^,^23^. In addition, activation of AMPK was reported to facilitate TJ formation during Ca^2^ -induced TJ assembly^24^. Therefore, in the following experiments, we investigated the effects of activation of AMPK on the polymerization of claudins.

### Direct phosphorylation of the cytoplasmic tail of claudin-1 by AMPK

In GFPmCL1L cells, N-terminally GFP-tagged claudin1 migrated as a doublet by SDS-PAGE. We noticed that the upper band of GFP-claudin-1 decreased when treated with KN-93 or AICAR (Fig. 4D). This finding suggested that CaMKII inhibition led to post-translational modification of claudin-1. After activation of AMPK, the upper band of claudin-1 was also decreased, suggesting that the mobility shift of claudin-1 was not owed to the phosphorylation by AMPK (Fig. 4D).

### Inhibition of mono-ubiquitination of claudin-1 by AMPK

Next, we tested the possibility that the slower-migrating form of claudin-1 was owed to ubiquitination. Treatment with MG-132, an inhibitor of proteasome, increased the intensity of the upper band of GFP-claudin-1, supporting the idea that this upper band corresponds to mono-ubiquitinated claudin-1 (Fig. 4D). Similar to the effects of AICAR or KN-93 treatment, treatment with MG-132 induced spontaneous polymerization of GFP-claudin-1 in GFPmCL1L cells (Fig. S2).

To determine which residues are phosphorylated and mono-ubiquitinated in claudin-1, we generated a series of alanine mutants of the cytoplasmic tail of claudin-1 and examined their mobilities using SDS-PAGE (Fig. 4E). Only one mutant (T191A) displayed an upwards shift that corresponded to mono-ubiquitinated claudin-1, suggesting that this mutant is constitutively mono-ubiquitinated. Therefore, we hypothesized that claudin-1 is phosphorylated by AMPK at its Thr-191 residue. An *in vitro* phosphorylation assay using recombinant AMPK proteins and GST fusion proteins containing the cytoplasmic tail of claudin-1 confirmed this hypothesis (Fig. 4F).

Considering these findings, we reasoned that phosphorylation by AMPK might prevent the mono-ubiquitination of claudin-1. Mono-ubiquitinated claudin-1 was barely detectable in cells that were preincubated with AICAR prior to treatment with MG-132 (Fig. 4G), confirming that phosphorylation by AMPK prevents the mono-ubiquitination of this protein. In the COOH tail of claudin-1, the lysine residue (Lys-189) is located close to the Thr-191 residue. The K189R/T191A double mutant was no longer mono-ubiquitinated (Fig. 4H), suggesting that Lys-189 is the mono-ubiquitinated residue in claudin-1. In addition, an immunoprecipitation experiment confirmed that the K189R mutant of claudin-1 was not ubiquitinated (Fig. 4I). These findings suggest that membrane retention of claudin-1 is controlled by degradation via the ubiquitin-proteasome system and via phosphorylation. To prevent spontaneous polymerization of claudin-1, cells deplete excess claudin-1 using the ubiquitin-proteasome system. Phosphorylation of claudin-1 by AMPK prevents mono-ubiquitination of claudin-1, resulting in an increase in unpolymerized claudin-1 at the plasma membrane and subsequent surplus TJ formation.

### Claudin phosphorylation by AMPK in response to hyperosmotic stress

**Figure 5:**
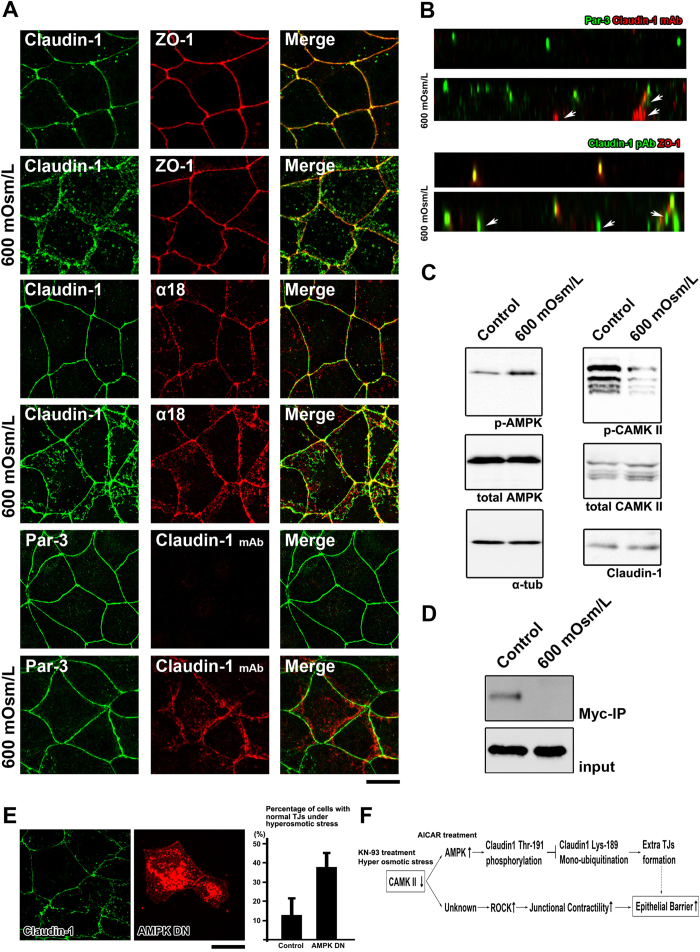
TJs enlarge as an adaptation response to hyperosmotic stress (**A**) CSG1 cells were treated with hyperosmotic medium for 2 hours and doubly stained with anti-claudin-1 pAb (green) and anti-ZO-1 mAb (red) (upper panels), anti-claudin-1 pAb (green) and α18 antibody (red) (middle panels) and/or anti-Par-3 pAb (green) and anti-claudin-1 mAb (Clone 2H10D10) (red) (lower panels). Scale bar, 20 μm. (**B**) Z-sections of CSG1 cells treated with hyperosmotic medium. (**C**) Immunoblotting of whole-cell lysates of CSG1 cells treated with normal osmotic medium or 600 mOsm/L medium for 2 hours with the indicated antibodies. (**D**) CSG1 cells stably expressing myc-ubiquitin were cultured in normal medium or hyperosmotic medium for 2 hours. The cell lysates were subjected to immunoprecipitation with anti-myc antibody, followed by immunoblotting with anti-claudin-1 antibody. (**E**) Over-expression of dominant negative AMPK2α suppressed the formation of extra TJs, even under hyperosmotic conditions. CSG1 cells were transfected with mCherry only as a control or mCherry-AMPKα2 K45M. Then, cells were cultured in hyperosmotic medium for 2 hours. Then, cells were fixed and stained for anti-claudin-1 pAb (green). The percentages were calculated as (the number of cells with normal TJs under hyperosmotic stress)/(the number of cells transfected with mCherry only or mCherry-AMPKα2 K45M). In each experiment, the total cell number was 100 (n = 3). Data are mean ± s.d.; *P < 0.05 (Student’s t-test). Scale bar, 10 μm. (**F**) The model of the molecular mechanism showing how KN-93 treatment enhances epithelial barrier function.

Finally, we examined whether the molecular mechanism revealed in this study is physiologically relevant. It was previously reported that hyperosmotic stress induces enlargement of TJ areas in epithelial cells^5^. However, the molecular mechanism behind this phenomenon remains unclear. When CSG1 cells were treated with 600 mOsm/L medium, claudin-1 staining indicated the formation of additional TJs (Fig. 5A,B). Similar to the additional TJs induced by CaMKII inhibition, undercoat proteins of TJs such as ZO-1 or Par-3 did not exist in these surplus TJ areas induced by hyperosmotic stress (Fig. 5A,B). The staining of α18 antibody and that of phosphorylated MLC2 was enhanced after hyperosmotic stress (Fig. 5A, and S3). We also confirmed surplus TJ formation under hyperosmotic conditions using anti-claudin-1 mAb (clone 2H10D10) and freeze-fracture electron microscopy (Fig. 5A,B and S4). CaMK II activity is reduced by treatment of cells with KN-93 or hyperosmotic medium. However, whereas KN-93 took several hours to induce excess TJ formation, enlargement of TJs occurred within 2 hours when cells were exposed to hyperosmotic stress. In future studies, it will be interesting to clarify the reasons underlying the differences between the times required for the induction of excess TJs by these two treatments.

We next examined whether phosphorylation of claudin-1 by AMPK is also involved in the response to hyperosmotic stress. We first confirmed that AMPK is activated and CaMK II is inactivated after exposure to hyperosmotic stress (Fig. 5C). Although we could not detect a band shift of endogenous claudin-1 by ubiquitination on SDS-PAGE, we did observe that endogenous mono-ubiquitinated claudin-1 was diminished after hyperosmotic stress by using a myc-ubiquitin pull-down assay (Fig. 5D). When AMPK activity was reduced by over-expression of a dominant negative form of AMPK, the surplus TJ formation was significantly inhibited under the hyperosmotic conditions (Fig. 5E).

From these results we concluded that phosphorylation of claudin-1 by AMPK is involved in the physiological adaptation to hyperosmotic stress. To enlarge TJs under hyperosmotic stress, cells supply claudin-1 by inhibiting mono-ubiquitination of claudin-1 through activation of AMPK. Future studies are necessary to reveal how hyperosmotic stress inhibits the activity of CaMKII.

## Discussion

In this study, we used chemical library screening to reveal CaMK II is involved in the regulation of posttranslational modification of claudin-1 and the modulation of epithelial barrier function. We found that CaMK II inhibition yielded two independent effects on epithelial cells. First, it enhanced junctional contractility, which up-regulated epithelial barrier function. Second, it enlarged the network of TJ strands (Fig. 5F). As previously discussed, the enlargement of TJ networks decreases transportation of various materials, including growth factors, across the epithelial sheets through the paracellular pathway^17^,^18^,^19^. Thus, the identification of CaMKII as a regulator of TJ functions and its functional analysis in this study are important first steps in expanding the study of the epithelial barrier and developing therapeutic strategies for diseases caused by defective epithelial barrier function, such as inflammatory bowel disease, asthma or atopic dermatitis.

In our study, KN-93 treatment induced the up-regulation of junctional contractility and increased barrier function. By contrast, other studies have shown that the activation of myosin light chain in MDCK II cells is associated with the opening of TJs and the down-regulation of barrier function^25^,^26^. We hypothesize that this discrepancy may arise from differences between the cell-lines used and/or the degree of phosphorylation of myosin light chain in each study. Further analyses are required to clarify this issue.

Although post-translational modifications to the C-terminal tails of claudins, such as phosphorylation, ubiquitination and SUMOylation, have been reported in previous studies^27^,^28^, the physiological significance of these modifications remained unknown. Similar to our observation of claudin-1, phosphorylation of claudin-16 at Ser-217 and claudin-2 at Ser-209 were reported to be involved in the localization and subsequent degradation of claudins by the lysosome^29^,^30^. However, the kinases that add these modifications and the functional importance of phosphorylation of claudin were not well understood. Therefore, it will be of interest to examine the involvement of AMPK in the phosphorylation of other members of the claudin family of proteins in the future studies.

As for the molecular mechanisms of degradation in lysosomes, it was reported that the E3 ubiquitin ligase LNX1p80 polyubiquitinates claudin-1^31^. However, we think that LNXp80 is not involved in the molecular mechanisms presented in this study. LNX1p80 localizes at TJs via direct binding to claudin-1 through its PDZ domain, and this interaction is essential for the ubiquitination of claudin-1; claudin-1 lacking the COOH terminal –YV was not ubiquitinated by LNX1p80. In our study, GFP-claudin-1, which cannot bind to LNX1p80 owing to steric hindrance produced by GFP, is still efficiently mono-ubiquitinated, and this mono-ubiquitination was blocked by the AMPK mediated phosphorylation. Therefore, poly-ubiquitination of claudin-1 catalyzed by LNX1p80 occurs at TJs, and LNX1p80 might control endocytic internalization of polymerized claudin-1 at TJs as proposed^31^. Considering that mono-ubiquitination of claudin-1 at the basolateral membrane is essential for the formation of extra TJs, we have begun to attempt to identify E3-ubuquitin ligase responsible for the ubiquitination of unpolymerized claudins at the basolateral membrane of epithelial cells.

## Materials and Methods

### Reagents

CSG1 cells, EpH4 cells and L fibroblasts were grown in Dulbecco’s modified Eagle’s medium supplemented with 10% fetal calf serum. The cDNA encoding full-length mouse claudin-1 was amplified by PCR, fused to the sequence for EGFP and ligated into the pCAGGS-neo vector.

The chemical library was obtained from SCADS (Screening Committee of Anticancer Drugs) of Japan. The following primary antibodies were used for the immunofluorescence microscopy and immunoblotting: rabbit anti-claudin-3 pAb (Zymed), rabbit anti-claudin-1 pAb, mouse anti-α tubulin mAb (Sigma), rabbit anti-AMPK antibody, rabbit anti-phospho-AMPK mAb, rabbit anti-CaMK II antibody, rabbit anti-phospho-CaMK II mAb, mouse anti-phospho MLC2 mAb (Cell Signaling Technology), mouse anti-claudin-1 mAb clone 2H10D10 (Life Technologies), rabbit anti-Par3 pAb (Millipore), mouse anti-ZO-1 mAb (T8-754) and α18 antibody (a generous gift from Dr. A. Nagafuchi).

The following chemical reagents were used for this study: KN-93 (Wako), AICAR (Sigma) and MG-132 (Peptide Institute. Inc.).

### *In Vitro* Kinase Assay

Recombinant active AMPK was purchased from Millipore. Glutathione S-transferase (GST)-tagged cytoplasmic tail of mouse claudin-1 (183 aa- 211 aa) were expressed in *Escherichia coli* and purified using Glutathione Sepharose Beads (GE Healthcare). Kinase reactions contained recombinant AMPK and GST-COOH-terminal tail of mouse claudin-1 substrates in 20 mM Tris-HCl (pH 7.5), 10 mM MgCl_2_, 25 μM ATP, and 15 μCi [γ-32P] ATP for 30 min at 37 °C. Proteins were separated by SDS-PAGE and visualized by autoradiography (Typhoon9500).

### Pull-down Assay

HEK293 cells were transfected with GFP-tagged claudin-1 or claudin-1 K189R and Myc-tagged ubiquitin. CSG1 cells were transfected with Myc-tagged ubiquitin alone. Cells were lysed with lysis buffer containing 20 mM Hepes (pH 7.4), 50 mM NaCl, 2 mM MgCl_2_, 1% Triton X-100, 2 mM DTT, 1 mM PMSF, 2 μg/mL leupeptin, and 0.25% aprotinin; lysates were cleared by centrifugation. Each supernatant was incubated with anti-Myc mAb (9E10)-conjugated protein G beads. After extensive washing with the lysis buffer, bound proteins were eluted with SDS-sample buffer. Samples were resolved by SDS/PAGE and electrophoretically transferred to a nitrocellulose membrane (Schleicher & Schuell). This membrane was incubated successively with anti-GFP mAb or anti-claudin-1 mAb which were visualized using Super Signal West Dura (Pierce).

### Freeze fracture electron microscopy

Freeze fracture electron microscopy was performed as described previously^32^. Confluent cells were fixed with 2.5% glutaraldehyde in phosphate buffer, rinsed in phosphate buffer, cryoprotected in 30% glycerol in phosphate buffer, and then frozen in liquid propane. Frozen samples were fractured at –110 °C and platinum-shadowed unidirectionally at an angle of 45° in a JFD-7000 apparatus (Jeol). Replicas with cells were immersed in household bleach and then mounted on copper grids after the cells were removed. They were examined using a Jeol 2000EX electron microscope.

### Immunofluorescence Microscopy

Immunofluorescence microscopy was performed as described previously^33^. In brief, cells cultured on coverslips were fixed with 3% formalin in PBS for 10 min at room temperature (RT), treated with 0.2% Triton X-100 in PBS for 5 min, and then washed with PBS. Blocking was done by incubating the fixed cells with 5% BSA in PBS for 30 min at RT. After the antibodies were diluted with the blocking solution, the cells were incubated at RT for 1 h with the primary antibody and for 30 min with the secondary antibody. For actin staining, Alexa Fluor 488 phalloidin (Life Technologies) was added to the secondary antibody.

Specimens were observed at RT with a confocal microscope (LSM700; Carl Zeiss MicroImaging, Inc.) equipped with a Plan-APO (63/1.40 N.A. oil-immersion) objective with appropriate binning of pixels and exposure time. The images were analyzed with ZEN 2012 (Carl Zeiss MicroImaging, Inc.).

## Additional Information

**How to cite this article**: Shiomi, R. *et al*. CaMKII regulates the strength of the epithelial barrier. *Sci. Rep*. **5**, 13262; doi: 10.1038/srep13262 (2015).

## Supplementary Material

Supplementary Information
