# Supplementary material for: CaMKII regulates the strength of the epithelial barrier
Source: Sci Rep. 2015 Aug 18;5:13262. doi: 10.1038/srep13262 (PMC4539604; doi:10.1038/srep13262)
Supplement: Supplementary Information [file srep13262-s1.pdf]

Supplementary Figures

CaMKII regulates the strength of the epithelial barrier

Ryo Shiomi, Kenta Shigetomi, Tetsuichiro Inai, Masami Sakai and Junichi Ikenouchi

Figure S1

Expansion of TJ strands in epithelial cells treated with KN-93

(A) CSG1 cells were doubly stained with anti-claudin-1 pAb (green) and ZO-1 mAb (red). The confocal xy sections shown are at the levels of TJs. The level of Z1 is the most apical region of TJs and the level of Z3 is the most basal side of TJs judging from the staining of ZO-1. Scale bar, 20 µm.

(B) CSG1 cells were treated with 10 µM KN-93 for 6 hours and doubly stained with α18 antibody (green) and anti-claudin-1 pAb (red). In KN-93 treated cells, the staining of clauidn-1 can be seen in the basal xy sections. Scale bar, 20 µm.

Figure S2

Effects of AIP, AICAR and MG-132 on the formation of TJs

(A) GFPmCL1cells cells (upper panel) or CSG1 cells (lower panel) were treated DMSO or with 1 µM Autocamide-2-related Inhibitory Peptide (AIP) for 6 hours. CSG1 cells were fixed and stained with anti-claudin-1 pAb. Scale bar, 20 µm.

(B) CSG1 cells were cultured in Transwell chambers and treated with DMSO (control) or 1 mM AICAR for 24 hours, then analyzed for their TER.

The TER values in this experiment were as follows: control 542.0±33.2 **Ω**/cm^2, 1 mM AICAR 514.0±41.0 **Ω**/cm^2.

(C) CSG1 cells were treated with 1 mM AICAR for 24 hours and doubly stained with α18 antibody (green) and anti-claudin-1 pAb (red). Scale bar, 20 µm.

(D) GFPmCL1cells were treated with 10 μM MG-132 for 8 hours. Scale bar, 20 μm.

Figure S3

Hyperosmotic stress increases phosphorylation of MLC-2

Phosphorylation of MLC-2 was up-regulated throughout the cytoplasm under hyperosmotic stress. Scale bar, 10 μm.

Figure S4

Hyperosmotic stress increases TJ strands

Freeze-fracture images of TJ strands of EpH4 cells treated with 600 mOsm/L medium for 2 hours. Scale bar, 200 nm.

A

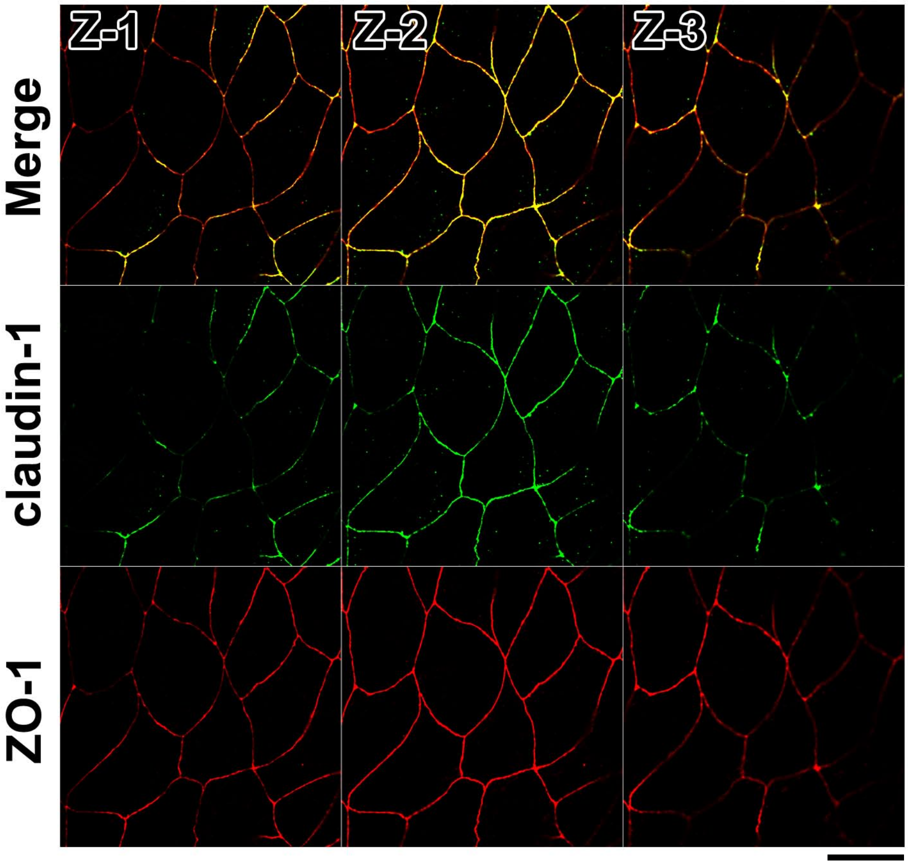

B

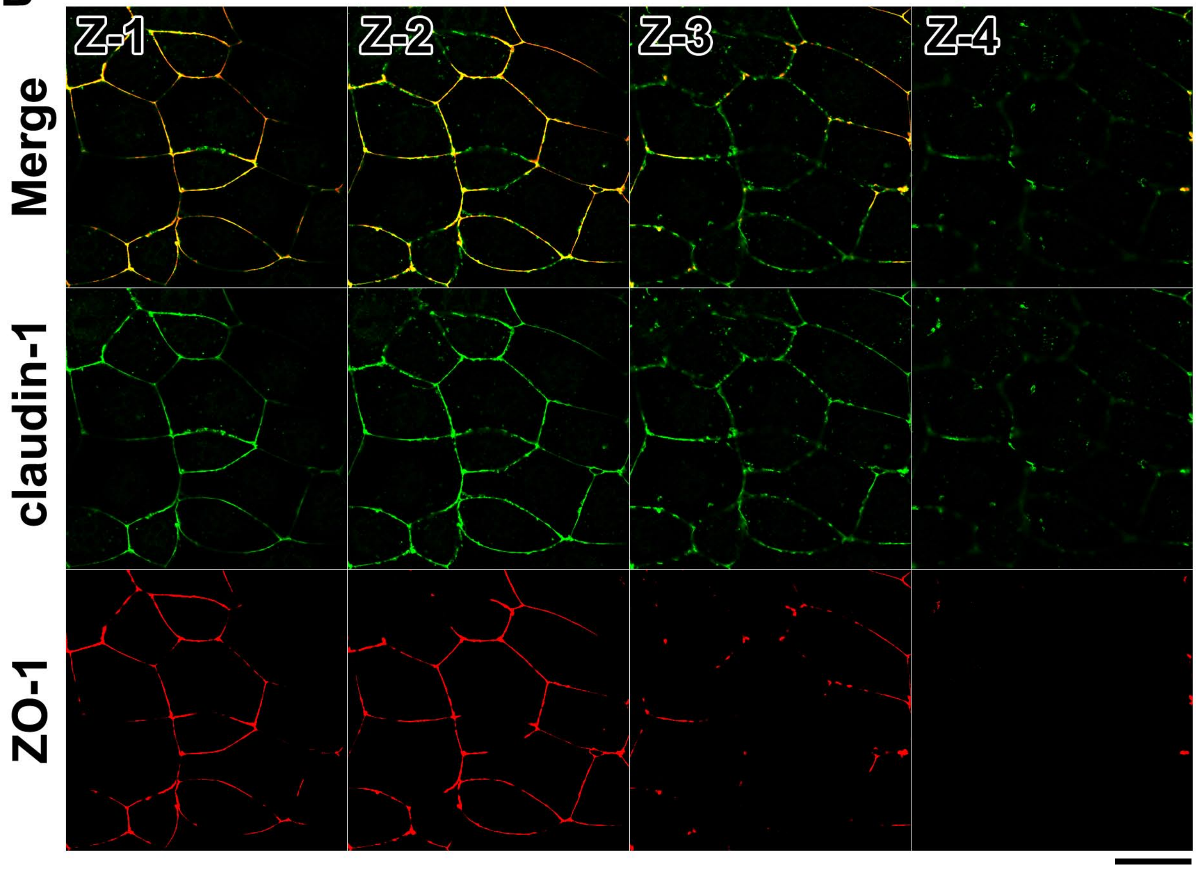

Figure S1 Shiomi et al.

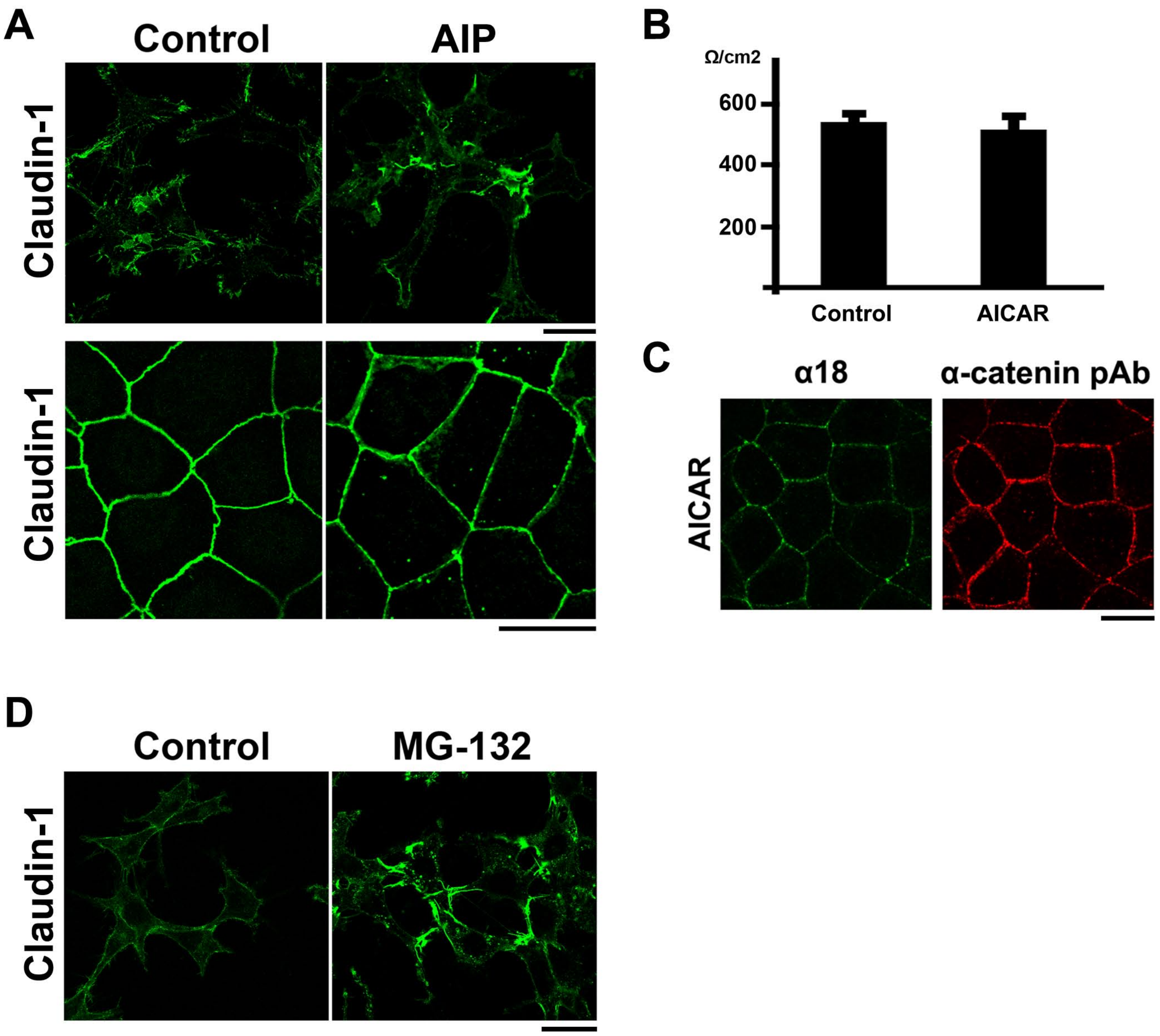

Figure S2 Shiomi et al.

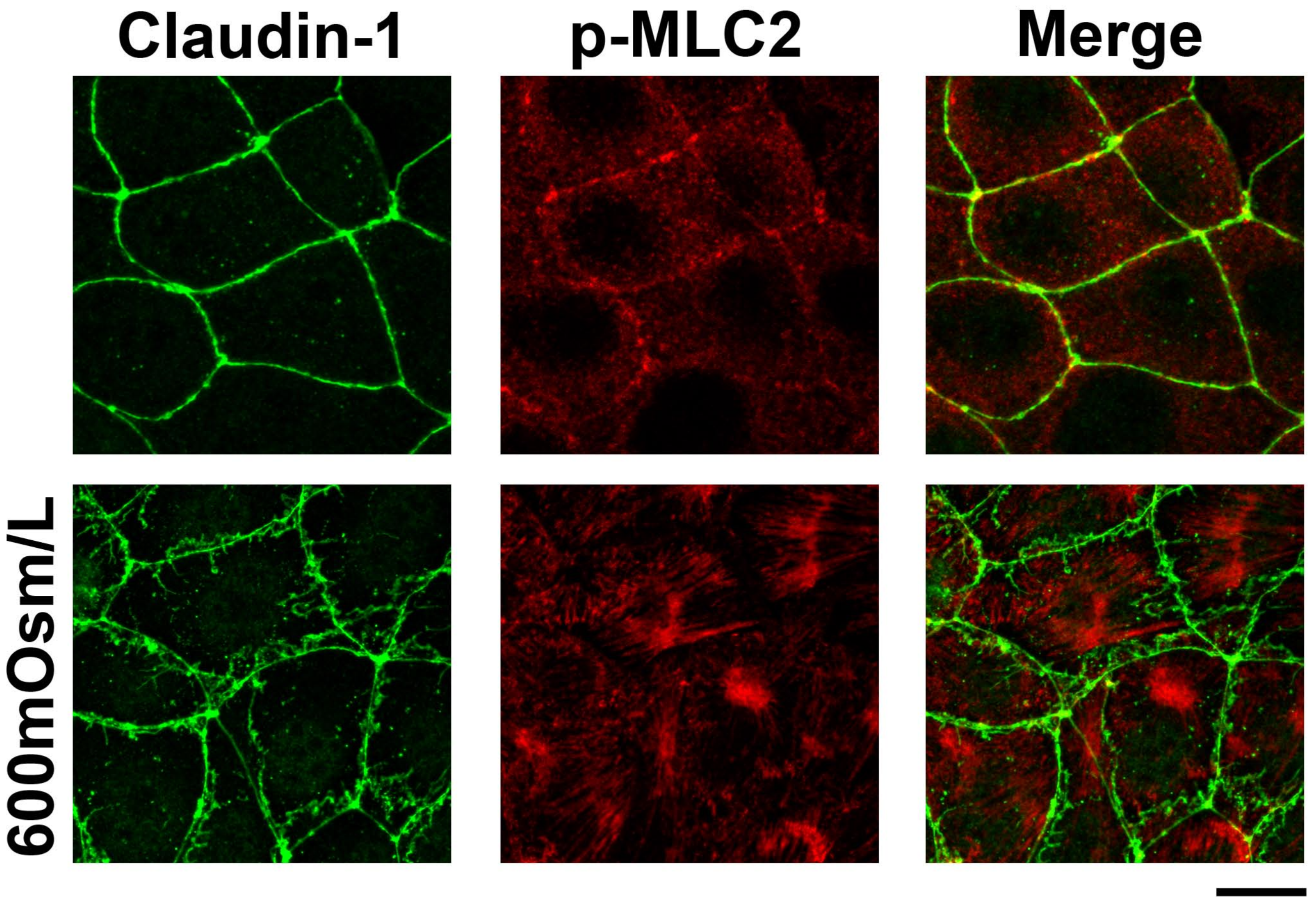

Figure S3 Shiomi et al.

600 mOsm/L

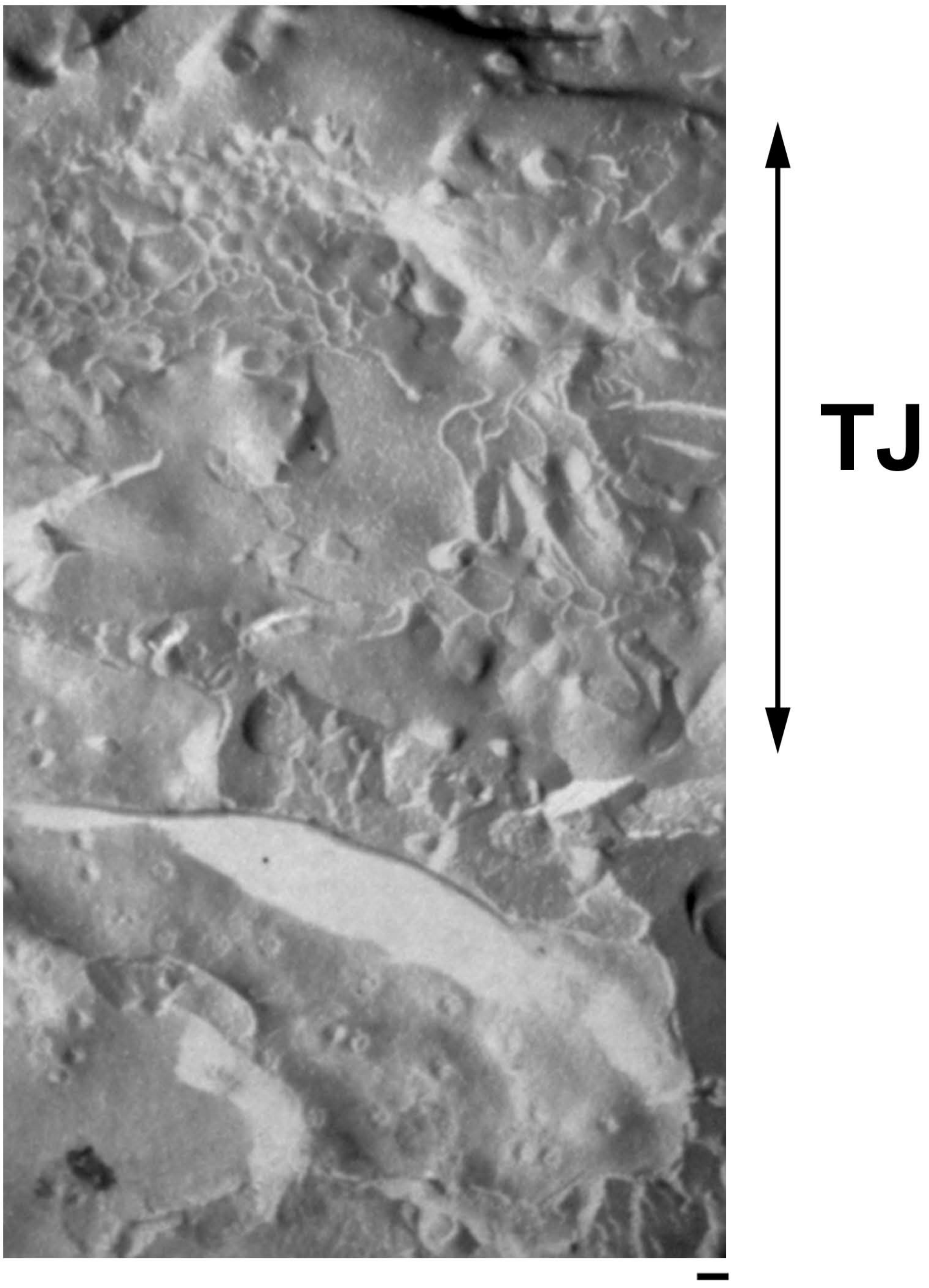

Figure S4 Shiomi et al.
